# Phenolic Profile, Antioxidant and Anti-Proliferative Activities of Methanolic Extracts from *Asclepias linaria* Cav. Leaves

**DOI:** 10.3390/molecules25010054

**Published:** 2019-12-23

**Authors:** José Alejandro Sánchez-Gutiérrez, Dafné Moreno-Lorenzana, Dioselina Álvarez-Bernal, Jacobo Rodríguez-Campos, José Roberto Medina-Medrano

**Affiliations:** 1 Instituto Politécnico Nacional, Centro Interdisciplinario de Investigación para el Desarrollo Integral Regional Unidad Michoacán, Jiquilpan 59510, Michoacán, Mexico; jasg94_7@hotmail.com (J.A.S.-G.); dalvarezb@ipn.mx (D.Á.-B.); 2 CONACYT-Instituto Nacional de Pediatría, Coyoacán 04530, Ciudad de México, Mexico; dmoreno@conacyt.mx; 3 Unidad de Servicios Analíticos y Metrológicos, Centro de Investigación y Asistencia en Tecnología y Diseño del Estado de Jalisco A.C. (CIATEJ), Guadalajara 44270, Jalisco, Mexico; jarodriguez@ciatej.mx; 4 CONACYT - Instituto Politécnico Nacional, Centro Interdisciplinario de Investigación para el Desarrollo Integral Regional Unidad Michoacán, Jiquilpan 59510, Michoacán, Mexico

**Keywords:** scavenging activity, phenolic compounds, antioxidant activity, ultra-performance liquid chromatography (UPLC), cytotoxic activity, *Asclepias linaria*

## Abstract

*Asclepias linaria* Cav. (Apocynaceae) is a shrubby plant endemic of Mexico which has been used in traditional medicine. However, the bioactive potential of this plant remains unexplored. In this study, the phenolic composition, antioxidant, and cytotoxic activities of *A. linaria* leaves were determined. In order to estimate the phenolic composition of the leaves, the total phenolic, flavonoid, and condensed tannins contents were determined. Furthermore, the antioxidant activity was measured by the scavenging activity of the 2,2-diphenyl-1-picrylhydrazyl (DPPH^•^) and 2,2′-azino-bis[3-ethylbenzothiazoline-6-sulphonic acid] (ABTS^•+^) radicals and the total antioxidant capacity. The phenolic compounds identified in the *A. linaria* leaves by ultra-performance liquid chromatography coupled to mass spectrometry (UPLC-MS) include phenolic acids, such as *p*-coumaric and ferulic acid, as well as flavonoids, such as rutin and quercetin. The leaves’ extracts of *A. linaria* showed a high scavenging activity of DPPH^•^ and ABTS^•+^ radicals (IC_50_ 0.12 ± 0.001 and 0.51 ± 0.003 µg/mL, respectively), high total antioxidant capacity values (99.77 ± 4.32 mg of ascorbic acid equivalents/g of dry tissue), and had a cytotoxic effect against K562 and HL60 hematologic neoplasia cells lines, but no toxicity towards the normal mononuclear cell line was observed. These results highlight the potential of *A. linaria* and could be considered as a possible alternative source of anticancer compounds.

## 1. Introduction

The Apocynaceae family have approximately 5000 species and 395 genera classified in five subfamilies [1,2]. The Asclepiadoideae subfamily is one of the five subfamilies included in the Apocynaceae family [3,4]. Within this subfamily is the American genus *Asclepias* (milkweeds) with about 150 species, distributed in North and South America [5]. The genus in the Americas is found in a wide range of habitats, from deserts to swamps, plains to shaded forests, and may represent a rapid ecological expansion [6]. Most of the species that constitute this genus are provided with highly-specialized lactiferous cells which synthesize a milky latex when they are injured [7]. Previous chemical investigations of *Asclepias* species have shown that different types of steroidal compounds, such as cardenolides, pregnanes, and androstanes, usually as glycosides, are the main metabolites of these plants [8,9,10,11]. However, flavonoid glycosides [12], megastigmane glycosides [13], triterpenes [8,14], conduritols, and conduritol glycosides [15] have been also isolated from these plants. 

Although *Asclepias* species are considered toxic, some of them are used in folk medicine as anthelmintic, analgesic, cardiotonic, and for the treatment of dermatological problems [16], cancer [8], pleuris, bronchitis [11], and asthma [14]. Such is the case of *Asclepias curassavica, Asclepias albicans, Asclepias subulata, Asclepias quinquedentata*, and *Asclepias linaria*. The species *A. linaria* Cav. is a plant endemic to Mexico which has been used in traditional medicine [16]. However, as far as we know, the bioactive potential of this plant remains unexplored. Therefore, the aim of the present work was to investigate the phenolic content and composition, antioxidant, and cytotoxic activity of *A. linaria* leaves from different populations.

## 2. Results and Discussion

### 2.1. Total Phenolic, Flavonoid, and Tannin Contents

In order to estimate the phenolic composition of the methanolic extracts of *A. linaria* leaves, the total phenolic, flavonoid, and condensed tannins contents were determined. The total phenolic contents in leaf extracts from wild populations of *A. linaria* were shown in a range from 7.17 ± 0.46 to 10.50 ± 0.27 milligrams of gallic acid equivalents per gram of dry tissue (mg GAE/g DT). Populations 3, 4, and 7, extracted with 50% methanol (P3-M50, P4-M50, and P7-M50), showed the highest phenolic contents, while Population 1 extracted with 50% methanol (P1-M50) obtained the lowest content, differences that were statistically significant (Table 1). A tendency can be observed in the extracts from *A. linaria* analyzed in the present work, since the highest total phenolic contents were observed when 50% methanol was used, which indicates that the solvent combination methanol-water was more effective in the extraction of phenolic compounds than the individual use of the methanol solvent. The mean total phenolic content value of the extracts of *A. linaria* leaves analyzed in this study was 9.17 mg GAE/g DT. Previous studies reported a concentration of 9.95 mg GAE/g DT in ethanolic extracts of *A. linaria* leaves [17], and concentrations of 2.87 ± 0.1 and 11.79 ± 0.18 mg GAE/g DT, for aqueous and ethanolic (80% ethanol) extracts, respectively, from aerial parts of *Asclepias syriaca* [18], both similar to the values obtained by the methanolic extracts analyzed in this work.

**Figure 1:**
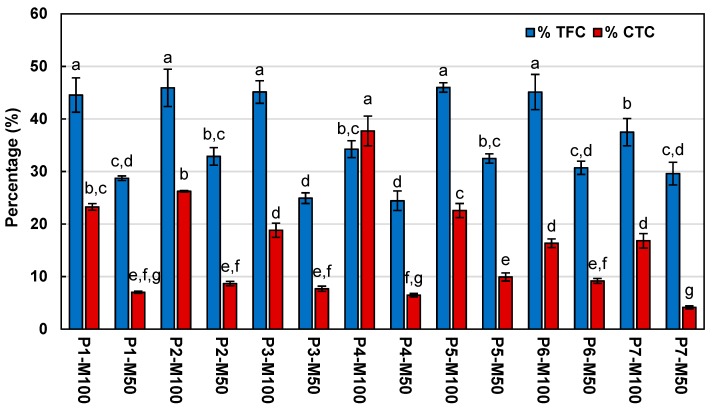
Average percentage of total flavonoid content (TFC) and condensed tannins content (CTC) from the total phenolic content (TPC) of methanolic extracts from *Asclepias linaria* leaves. Values are expressed as the mean ± standard deviation of three repetitions. Values with different letters indicate significant differences (Tukey, *p* < 0.05).

Regarding the total flavonoids content in the methanolic extracts of *A. linaria* leaves, significant difference between samples was shown, since the highest content was observed in Population 5 extracted with 100% methanol (P5-M100), with 4.17 ± 0.31 mg of catechin equivalents (CE)/g DT, while the lowest content was observed in Population 4 extracted with 50% methanol (P4-M50), with 2.56 ± 0.26 mg CE/g DT. The above values represent 45.9 and 24.4%, respectively, of the total phenolic content (Figure 1).

As seen in Table 1, the total condensed tannins content was shown in a range from 0.43 ± 0.03 to 3.16 ± 0.16 mg CE/g DT. Population 4 extracted with methanol (P4-M100), showed the highest condensed tannins contents, while Population 7 extracted with 50% methanol (P7-M50) obtained the lowest content, differences that were statistically significant according to the analysis. As with the total flavonoid content, a clear tendency was observed, since a significantly highest tannins content was shown by the samples extracted with 100% methanol, followed by samples extracted with 50% methanol. 

According to the analysis, a no clear correlation was observed between the different determinations of phenolic content, since the extracts that shown the highest total phenolic contents did not obtain the highest total flavonoid and condensed tannin contents. However, the highest percentages of flavonoids and condensed tannins were obtained in the samples extracted with 100% methanol (Figure 1), behavior that could be related to the effect of the solvent on the extraction of phenolic compounds, since, as mentioned above, the samples extracted with 100% methanol showed the highest contents of total flavonoids and condensed tannins.

### 2.2. Phenolic Compound Analysis by Ultra-Performance Liquid Chromatography Coupled to Mass Spectrometry (UPLC-MS)

The results of the phenolic profiles of the methanolic extracts from *A. linaria* leaves obtained by UPLC-MS revealed the presence of four phenolic compounds: two phenolic acids derived from cinnamic acid (*p*-coumaric acid and ferulic acid) and two flavonoids (rutin and quercetin). The data obtained did not indicate the presence of apigenin, naringin, catechin, vanillin, chlorogenic, cinnamic, gallic, and caffeic acid (Table 2). UPLC-MS chromatographic data showed variations on the phenolic composition and concentrations between the *A. linaria* populations analyzed in the present work. In addition to this, it was observed that the concentration and phenolic composition of the extracts were notoriously affected by the extraction solvent used, since the extracts with 100% methanol showed, in most cases (with exception of quercetin), higher individual concentration of the phenolic compounds was detected. The concentrations of the phenolic compounds identified in the 100% and 50% methanolic extracts from *A. linaria* leaves are summarized in Table 2. 

As seen in Table 2, *p*-coumaric acid was identified in two of the seven populations extracted with 100% methanol (P4-M100 and P5-M100), and it was not identified in any of the evaluated populations. This behavior (presence or absence of *p*-coumaric acid) may be due to the solubility and polar nature of this compound, showing greater affinity to the solvent 100% methanol. Other authors reported the presence of vanillic, syringic and *p*-coumaric acid, in aqueous extracts of aerial parts from *A. syriaca* [18], while resorcylic, protocatechuic, *p*-hydroxybenzoic, syringic, caffeic, coumaric, ferulic and chlorogenic acid and the flavonoid vanillin, in ethanolic extracts of leaves and flowers of the same species [19]. The flavonoid rutin, compound present in all the analyzed extracts, showed to be the majority compound, since it obtained percentages of up to 90% and 100% (P6-M100 and P6-M50, respectively) with respect to the total phenolic composition. Overall, extracts from Population 7 (P7-M100 and P7-M50) showed a quantitative phenolic composition superior to the rest, which was statistically significant according to the analysis (*p* < 0.05). The correlation analysis indicated a significant positive correlation between rutin and ferulic acid contents (*R* = 0.881, *p* < 0.01) and between *p*-coumaric acid and the total condensed tannins content (*R* = 0.743, *p* < 0.01) of the extracts.

The presence of phenolic compounds has not been previously reported for the *A. linaria* species analyzed in this work. Identification of this phenolic compounds in *A. linaria* extracts should be helpful for further separation and bioactivity study. 

### 2.3. Antioxidant Activity

Phenolic compounds have shown to possess antioxidant properties. In this work, *A. linaria* leaves extracts showed scavenging activity of ABTS^•+^ and DPPH^•^ radicals as well as relevant antioxidant capacity. 

As seen in Table 1, a significant difference between samples was observed. The extracts from Populations 1 and 3 extracted with 50% methanol (P1-M50 and P3-M50) exhibited the lowest IC_50_ values and thus significantly highest scavenging activity of ABTS^•+^ radical. Regarding the scavenging activity of DPPH^•^ radical, in the same way as with ABTS^•+^ radical, the extract from Population 1 extracted with 50% methanol (P1-M50) shown the lowest values IC_50_ (Table 1). Interestingly, P1-M50 extract showed a high phenolic content but a low concentration of flavonoids and condensed tannins (Table 1). Despite the above, their main constituents were quercetin and rutin flavonoids (Table 2), which have a high free radical scavenging activity reported, behaving as the strongest O_2_ radical scavengers [20,21]. 

The significantly higher scavenging activity of ABTS^•+^ and DPPH^•^ radicals were shown by samples of *A. linaria* extracted with 50% methanol, and the samples that showed the lowest scavenging activity of ABTS^•+^ and DPPH^•^ radicals were the samples extracted with 100% methanol, behavior that could be related to the effect of the solvent on the extraction of phenolic compounds, since as mentioned above, the samples extracted with the hydromethanolic combination showed the highest contents of total phenolic compounds. Additionally, a strong correlation was observed between the total phenolic content with the IC_50_ values of ABTS^•+^ radical (*R* = −0.723, *p* < 0.01) indicating that increasing quantity of total phenolics leads to a quantitative decrease of the IC_50_ values of the extracts and thus significantly higher scavenging activity of ABTS^•+^ radical. Otherwise, unclear correlations were shown between the phenolic contents of the *A. linaria* extracts or their phenolic compounds detected by UPLC-MS with the scavenging activity of DPPH^•^ radical. Regarding the scavenging activity of ABTS^•+^ and DPPH^•^ radicals of the standard rutin used as a positive control, IC_50_ values of 0.062 ± 0.001 and 0.28 ± 0.001 mg/mL, respectively, were observed. 

The analysis of the total antioxidant capacity of the methanolic extracts from leaves of seven populations of *A. linaria* showed values in a range of 17.96 ± 1.47 to 99.77 ± 4.32 mg of ascorbic acid equivalents (AAE)/g DT (Table 1). The extract from Population 1 extracted with 50% methanol (P1-M50) shown the lowest antioxidant capacity, while the extracts of *A. linaria* from Populations 6 and 7, extracted with 100% methanol (P6-M100 and P7-M100) exhibited the highest antioxidant capacity, with 99.77 ± 4.32 and 95.49 ± 4.32 mg AAE/g DT, respectively. A marked effect of the extraction solvent on the total antioxidant activity was observed, since as with the total flavonoid and tannins contents, a significantly highest antioxidant capacity was shown by the samples extracted with 100% methanol. The correlation analysis did not show a clear correlation between the phenolic, flavonoid, and condensed tannins contents with the total antioxidant capacity, however, the antioxidant capacity will not always depend on the concentration of phenolic compounds, but rather on the type of antioxidant compound, the amount of hydroxyl groups and their position within the antioxidant molecule they constitute [20]. In that sense, both extracts (P6-M100 and P7-M100) revealed high individual concentrations of ferulic acid, rutin and quercetin, and, specifically, the highest concentrations of ferulic acid and rutin (P7-M100), this based on the phenolic profiles obtained by UPLC-MS, which are shown in Table 2. In accordance with the above, moderate correlations were shown between individual concentrations of ferulic acid and rutin with total antioxidant capacity (*R* = 0.680, *p* < 0.01 and *R* = 0.723, *p* < 0.01, respectively). The above can help to deduce the remarkable antioxidant capacity that the extracts with the highest concentration of these two flavonoids showed. In addition, some authors establish that the antioxidant capacity of ferulic acid may become similar to that of ascorbic acid (vitamin C) [22], so this could also influence the increase in the antioxidant capacity of the extracts of *A. linaria* from these populations. 

Considering our results, we can hypothesize that the presence of flavonoids in *A. linaria* may be responsible for their radical scavenging activity and antioxidant capacity.

### 2.4. Cytotoxic Activity

The cytotoxic effect of the foliar methanolic extracts of *A. linaria* against three cell lines was determined, of which two were hematologic neoplasia cells lines (K562 and HL60) and, as non-cancer, normal mononuclear cells (NMC). Furthermore, a positive control treated with 2.5 μM imatinib was used. In K562 cells treated with imatinib a 99.60 ± 0.06% inhibition on cell viability was observed. The values of median inhibitory concentration (IC_50_) of the extracts of *A. linaria* obtained are summarized in Table 3. 

As observed in Table 3, based on the IC_50_ values, the extracts that had the greatest effect against K562 and HL60 hematologic neoplasia cell lines, correspond to P2-M50 and P7-M100. As mentioned earlier, although the P7-M100 extract showed moderate concentrations of total phenols, flavonoids and condensed tannins (Table 1), it also showed the highest individual concentrations of ferulic acid and rutin, as well as moderate concentrations of quercetin (Table 2). As reported in the literature, these phenolic compounds could have a direct relationship with the anti-proliferative and cytotoxic effect observed against K562 and HL60 cells through various mechanisms, which are: inhibition of the enzyme ribonucleotide reductase (responsible for reducing deoxyribonucleotides to ribonucleotides); BCR/ABL tyrosine kinase protein inhibition, NF-κB and COX-2; induction of cell apoptosis by activation of caspases 9 and 3; increase in Bax protein (pro-apoptotic protein); arrest of tumor growth or even induction of apoptosis by intracellular increase of reactive oxygen species (ROS) [23,24,25]. These effects will depend on the dose and concentration of phenolic compounds present in the extracts. In accordance with the above, moderate correlations were shown between individual concentration of ferulic acid with IC_50_ values of the extracts against HL60 cell line (*R* = −0.611, *p* < 0.01). 

Regarding the effect of quercetin and rutin flavonoids, these compounds are considered as one of the most active against cancer, even using low concentrations, since the characteristic mechanisms of these compounds are: condensation of nuclear chromatin; fragmentation of genetic material (DNA); arrest of the cell cycle; enzyme tyrosine kinase inhibition and induction of apoptosis in cells on G_1_ and S phase of the cell cycle [10,26,27]. Similarly, other authors mention that the combination of two or more of these mechanisms may be responsible for the chemotherapeutic or preventive nature of polyphenolic compounds [28]. These mechanisms have been reported in different cell lines, including K562. The observed cytotoxic effects could be related to the presence of ferulic acid, rutin, and quercetin in the *A. linaria* leaves extracts.

Table 3 also shows the results of growth inhibition of NMC as well as the SI obtained. The extracts that showed the least effect (which is what is desired in this case) were P3-M50 and P7-M50, with an IC_50_ values of 187.30 ± 43.58 and 244.9 ± 96.16 µg/mL, respectively. To the author’s best knowledge, there are no previous reports on the cytotoxic effects of *A. linaria* extracts. 

### 2.5. Reactive Oxygen Species (ROS)

**Figure 2:**
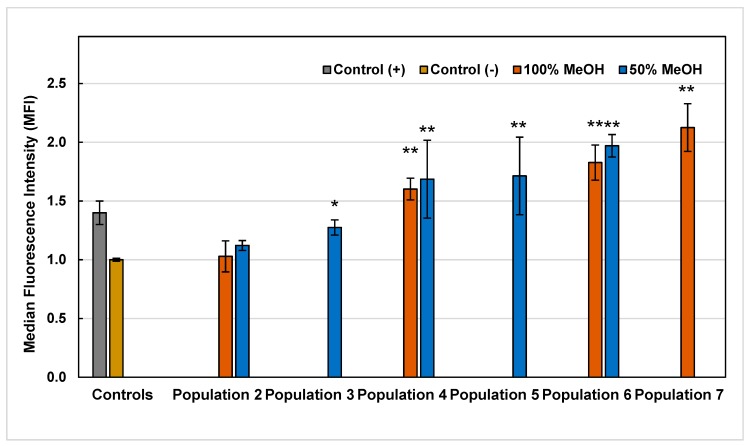
Intracellular ROS production values generated by the exposure of HL60 cells to 50% and 100% methanolic extracts of *A. linaria* after 1 h. Values are expressed as the mean ± standard deviation of three repetitions. (*) Indicates significant statistical difference from the control according to Dunnett’s test (*p* < 0.05), (**) *p* < 0.01.

**Figure 3:**
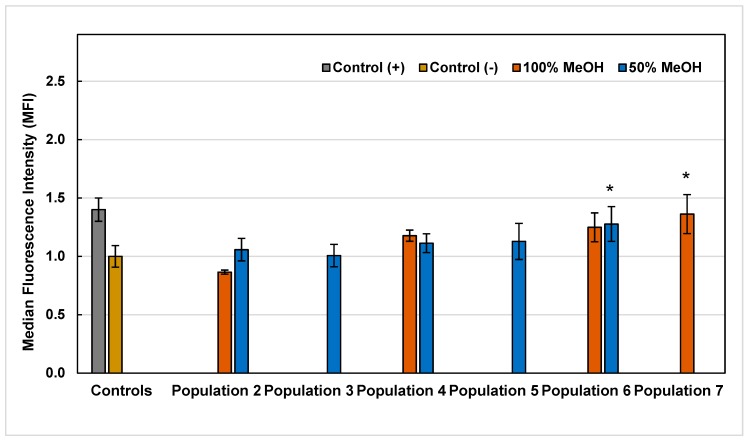
Intracellular ROS production values generated by the exposure of HL60 cells to 50% and 100% methanolic extracts of *A. linaria* after 3 h. Values are expressed as the mean ± standard deviation of three repetitions. (*) Indicates significant statistical difference from the control according to Dunnett’s test (*p* < 0.05).

With the intention of elucidating and proposing a possible mechanism of action through which the methanolic extracts from *A. linaria* leaves induced cytotoxicity and affected proliferation in cancer cells, it was decided to evaluate the effect on intracellular production of ROS, caused by the exposure of HL60 cells to extracts at two different times: 1 and 3 h. According to the discrimination made, nine of the 14 extracts were selected for the ROS assay. The results of the intracellular production of ROS obtained are presented in Figure 2 and Figure 3.

As can be seen in Figure 2, after 1 h of the exposure of HL60 cells to the extracts, seven of the nine extracts evaluated had a significant pro-oxidant effect, since extracts from Population 3 to 7 increased the value of initial median fluorescence intensity (MFI) of control cells, generated by the baseline ROS that characterize HL60 cells (in a range from 0.03 to 1.13 MFI). Otherwise, it was observed with P2-M50 and P2-M100 extracts, since their ROS production was not statistically different from the control, therefore, they showed no pro-oxidant effect. On the other hand, 3 h after exposure, it was observed that only two of the nine extracts evaluated maintained the pro-oxidant effect (P6-M50 and P7-M100), the others were not statistically different from the control (Figure 3). The P6-M50 and P7-M100 extracts that maintained the pro-oxidant effect showed high concentrations of phenolic, flavonoid and tannin compounds in general (Table 1), and, specifically, for P7-M100 extract, the highest concentrations of rutin and quercetin (Table 2). Otherwise, it was observed in the P6-M50 extract, which did not report considerable concentrations of any of the 12 standards analyzed by UPLC-MS. Accordingly, the phenolic composition of this extract could be completely different from the other extracts analyzed in this study.

The observed results can be considered contradictory, since the main mechanism attributed to phenolic compounds is to function as antioxidant molecules, however, there are several investigations that indicate that phenolic compounds, specifically flavonoids such as rutin and quercetin, have the ability to behave as pro-oxidants, this depending on the concentration, structure, functional groups, and their location within the molecule, and most importantly, the conditions under which these compounds carry out the oxidation-reduction reaction [29,30,31,32,33,34]. The pro-oxidant capacity of these compounds will also depend on the presence of pyrogallol or catechol groups in these compounds, since they induce the production of highly cytotoxic hydrogen peroxide radicals (H_2_O_2_) [31,32]. In addition, this effect has been observed more frequently when the doses or concentrations of flavonoids are high, as in this case. Based on the above, it can be inferred that the extracts of *A. linaria* that showed higher concentrations of flavonoids, increase the possibility of behaving as pro-oxidants, this under the physiological conditions established by cancer cells.

The effect generated by the extracts of *A. linaria* analyzed in this study was considered partly beneficial, because the extracts showed cytotoxic effect and could be considered as a possible alternative for new research and source of anticancer compounds.

## 3. Materials and Methods 

### 3.1. Reagents and Standards

The Folin–Ciocalteu reagent, 2,2-diphenyl-1-picrylhydrazyl (DPPH^•^), 2,2′-azino-bis[3-ethylbenzothiazoline-6-sulphonic acid] (ABTS**^•+^**), potassium persulfate, ammonium molybdate, ascorbic acid, sodium carbonate, aluminum chloride, formic acid, methanol, and water (HPLC grade) were purchased from Sigma-Aldrich (St. Louis, MO, USA). Nylon membranes (0.22 µm, Millipore, Milford, MA, USA) were used for ultra-performance liquid chromatography coupled to mass spectrometry (UPLC–MS) analysis. Roswell Park Memorial Institute culture medium (RPMI-1640), fetal bovine serum (FBS), streptomycin/penicillin, fluorescence-activated cell sorting (FACS) Flow cytometry solution, 4′,6′-diamino-2-phenylindole (DAPI), 2′,7′-dichlorodihydrofluorescein diacetate (DCFDA), amphotericin B, and dimethylsulfoxide (DMSO) were purchased from Gibco (Grand Island, NY, USA). Ficoll-paque Plus was purchased from GE Healthcare Bio-Sciences (Uppsala, Sweden). Endothelial basal medium-2 (EBM-2) was purchased from Cambrex (Walkersville, MD, USA). Parthenolide was purchased from Abcam (Cambridge, MA, USA). Turk solution and trypan blue were purchased from Hycel (Estado de México, México). Imatinib was purchased from SelleckChem (Houston, TX, USA).

Caffeic acid, chlorogenic acid, cinnamic acid, ferulic acid, gallic acid, p-coumaric acid, apigenin, catechin, naringin, rutin, quercetin, and vanillin (>99%) (Sigma-Aldrich, St Louis, MO, USA). Standards were prepared as stock solutions in methanol. Working solutions of standards were obtained by diluting the stock solution in methanol to obtain concentrations ranging from 0.25–5 µg/mL. Working solutions of standards were stored in dark conditions at −18 °C.

### 3.2. Plant Material and Sample Preparation

*A. linaria* specimens were collected during the months of September and October 2017 at Fray Dominguez, Pajuacarán, Mexican state of Michoacán (20°5´58.999” N, 102°33´12.999” W, and 1827 m altitude). The specimens were authenticated by Dr. Monserrat Vázquez Sánchez, from Colegio de Postgraduados, Campus Montecillo, México. Voucher herbarium specimens were deposited at the herbarium of CIIDIR-IPN Unidad Michoacán, with herbarium number MVS29. Seven populations were collected. Leaves were separated from the plants and dried in an oven at 50 °C for 48 h. Then, the leaves were ground in a mortar until a fine powder was obtained. Subsequently, a sieve (number 60) was used in order to homogenize the particle size to 250 µm. Samples were stored at room temperature in dark conditions until they were used. 

The extraction of phenolic compounds was carried out using 1 g of dry milled tissue which was macerated in 20 mL of solvent (50% methanol, or 100% methanol, *v*/*v*) by agitation at 100 rpm using a shaking table in the dark at room temperature for 24 h. Subsequently, the extracts were centrifuged at 2722× *g* for 10 min at room temperature. The supernatant was recovered and filtered through Whatman No. 1 filter paper (pore size 11 µm) (Whatman International Ltd., Maidstone, UK) to form the crude extract. Aliquots of the extract were taken for the phenolic content and antioxidant determinations.

### 3.3. Determination of Total Phenolic, Flavonoid, and Tannin Contents

#### 3.3.1. Determination of Total Phenolic Content

The determination of total phenolic contents of the samples was carried out using the Folin–Ciocalteu reagent method with modifications [35]. Two hundred and fifty µL of methanolic extract were mixed with 2.5 mL of distilled water, followed by 125 µL of 1N Folin–Ciocalteu reagent, and stirred for 5 min. Finally, 375 µL of 20% (*w/v*) Na_2_CO_3_ solution was added and kept up in dark conditions for 2 h at room temperature. Absorbance was read at 760 nm using a PowerWave HT Microplate Spectrophotometer (BioTek Instruments, Inc., Winooski, VT, USA). Total phenolic contents were estimated using a gallic acid standard curve (A_760_ = 0.0027 [gallic acid] + 0.0211, *R^2^* = 0.9889), obtained using seven known concentrations (0.04–0.46 mg/mL) of the compound. Total phenolic content was expressed as milligrams of gallic acid equivalents per gram of dry tissue (mg GAE/g DT).

#### 3.3.2. Determination of Total Flavonoid Content

Total flavonoid content of each sample was determined by AlCl_3_ method previously reported, with slight modifications [36]. One mL of methanolic extract was added with 1 mL of 2% (*w/v*) solution of AlCl_3_·6H_2_O. Absorbance was measured after 10 min at 430 nm using a PowerWave HT Microplate Spectrophotometer (BioTek Instruments, Inc., Winooski, VT, USA). Total flavonoid contents were calculated using a catechin standard curve (A_430_ = 0.0326 [catechin] − 0.0011, *R^2^* = 0.9900) obtained using seven concentrations of catechin (0.05–0.5 mg/mL). Flavonoid contents were expressed as milligrams of catechin equivalents per gram dry tissue (mg CE/g DT).

#### 3.3.3. Determination of Condensed Tannins

The vanillin-H_2_SO_4_ methodology was used to determine procyanidins contents in the extracts [37]. A total of 250 µL of each sample were reacted with 250 µL of 1% vanillin (*w/v*, dissolved in methanol) followed by 250 µL of the 25% sulfuric acid solution (*v/v*, dissolved in methanol) and incubated at a temperature of 30 °C for 15 min. Absorbance was read at 500 nm using a PowerWave HT Microplate Spectrophotometer (BioTek Instruments, Inc., Winooski, VT, USA). To estimate the concentration of tannins, a calibration curve with catechin (A_500_ = 1.5821 [catechin] + 0.0094, *R^2^* = 0.9960) was performed at different concentrations (0.01–0.25 mg/mL). Condensed tannins contents were expressed as milligrams of catechin equivalents per gram dry tissue (mg CE/g DT).

### 3.4. Phenolic Compounds Analysis by Ultra-Performance Liquid Chromatography Coupled to Mass Spectrometry (UPLC-MS)

#### 3.4.1. Sample Preparation for Phenolic Compounds Determination by UPLC-MS

The extracts (2.5 mL) were transferred to a 10 mL glass vial and taken to full dryness under a gentle stream of nitrogen at 50 °C using a TurboVap Classic LV (Biotage, Charlotte, NC, USA) at constant pressure (12 psi for 1h and 16 min). Each extract was resuspended individually in 2 mL of distilled water and frozen at −20 °C for 48 h. Subsequently, the extracts were lyophilized using a Labconco freeze dryer, model 77530 (Labconco, Kansas City, MO, USA) at −56 °C for 72 h and under high vacuum conditions (0.014 mBar). Once dried, the extracts were stored under refrigeration at 4 °C protected from light.

#### 3.4.2. Identification and Quantification of Phenolic Compounds by UPLC-MS

An Acquity I-class UPLC system (Waters, Milford, MA, USA) was used for the analysis, and consisted of a quaternary pump (QSM), a flow-through-needle (FTN) cooled autosampler, a column oven, and a sample organizer, equipped with a Waters BEH C18 column (100 mm × 2.1 mm, 1.7 µm) (Waters, Dublin, Ireland), and was coupled to a Xevo TQ-S micro Tandem Mass Spectrometer (Waters, Milford, MA, USA). Dry extracts were resuspended individually in absolute methanol and 20 μL of extract were injected into the system. The column temperature was maintained at 30 °C. The mobile phases were 0.3% formic acid (A) and methanol (B), with a flow rate of 0.3 mL/min. The elution gradient was as follows: 90% A as initial condition, 30% A at 11 min, and 90% A at 15 min. The conditions of the micro TQ-S were as follows: 3 kV capillary voltage, the ionization source was in electrospray mode (ESI^-^ and ESI^+^), the desolvation temperature was 500 °C, the desolvation gas (nitrogen) flow was set at 1000 L/h and the source temperature was 150 °C. Measurements were performed in multiple reaction monitoring (MRM) mode. The selection of MRM transitions and optimization of product ions was performed by infusion of individual standard solutions of all the analytes. Fractional MRM was conducted to ensure the number of acquisitions at least 20, making the dwell time reach a maximum for each MRM transition. Table 4 summarizes the conditions for all MRM transitions, including the retention time (*t*_R_). The standards used were: caffeic acid, chlorogenic acid, cinnamic acid, ferulic acid, gallic acid, *p*-coumaric acid, apigenin, catechin, naringin, rutin, quercetin, and vanillin. Quantification of the phenolic compounds was carried out using a calibration curve in the range of 0.25–5 µg/mL. The concentration was expressed as micrograms per milliliter (µg/mL) of extract. The sum of all individual phenolic compounds identified was expressed as total phenolic compounds.

### 3.5. Antioxidant Activity

#### 3.5.1. DPPH^•^ Free Radical Scavenging Assay

The determination of free radical scavenging activity was performed using the DPPH^•^ method previusly described with modifications [38]. For this method, a 24 µM ethanol solution of DPPH^•^ was prepared. To determine the scavenging activity, 90 µL of DPPH^•^ reagent were mixed with 10 µL of extract (concentration gradually increased, 0.025–0.250 mg/mL) and they were incubated at room temperature for 10 min. After incubation, the absorbance was measured at 523 nm using a PowerWave HT Microplate Spectrophotometer (BioTek Instruments, Inc., Winooski, VT, USA). 

The scavenging effect of DPPH^•^ was measured using the formula:

(1) $${\mathrm{DPPH}}^{•}\;\mathrm{scavenging}\;\mathrm{effect}\;(\%)\;=\;[(A_{\mathrm{control}}\;-\;A_{\mathrm{sample}}\;)\;/\;A_{\mathrm{control}}]\;\times \;100$$

where *A*_control_ is the absorbance of the control (DPPH^•^ solution), and *A*_sample_ is the absorbance of the test sample (DPPH^•^ solution plus 10 µL of extract). The scavenging effect was expressed as the median inhibitory concentration (IC_50_) in milligrams per milliliter (mg/mL). The IC_50_ represents the extract concentration needed to reduce by 50% the initial DPPH^•^ absorbance. IC_50_ was determined using linear regression. Rutin (0.05–0.5 mg/mL dissolved in methanol) was used as a positive control.

#### 3.5.2. ABTS^•+^ Radical Scavenging Assay

Antioxidant activity of methanolic extracts were evaluated using ABTS^•+^ radical scavenging assay, following methodology previously described [39]. Briefly, ABTS^•+^ was dissolved in distilled water to 7 mM concentration. ABTS^•+^ radical cations were produced by reacting 1 mL of ABTS^•+^ stock solution with 17.6 µL of 140 mM potassium persulfate and allowing the mixture to stand in the dark at room temperature for 12 h before use. The ABTS^•+^ solution was diluted with deionized water to obtain an absorbance of 0.70 (±0.01) at 734 nm. After addition of 50 µL of diluted ABTS^•+^ radical solution to 50 µL of the sample (*A. linaria* leaves extracts), the absorbance was registered at 734 nm after 6 min using a PowerWave HT Microplate Spectrophotometer (BioTek Instruments, Inc., Winooski, VT, USA). The assay was performed with nine extract concentrations (0.125–1.250 mg/mL, dissolved in methanol). The scavenging effect percentage was calculated using the formula:

(2) $${\mathrm{ABTS}}^{•+}\;\mathrm{scavenging}\;\mathrm{effect}\;(\%)\;=\;[(A_{\mathrm{blank}}\;-\;A_{\mathrm{sample}}\;)\;/\;A_{\mathrm{blank}}]\;\times \;100$$

where *A*_blank_ represents the absorbance of the blank (ABTS^•+^ solution plus 50 µL of corresponding solvent), *A*_sample_ is the absorbance of the test sample (ABTS^•+^ solution plus 50 µL of extract). The extract concentration (mg/mL) that provided a 50% inhibition of the ABTS^•+^ radical (IC_50_) was calculated from a graphic built with inhibition percentage versus extract concentration. Rutin (0.006–0.15 mg/mL dissolved in methanol) was used as a positive control.

#### 3.5.3. Total Antioxidant Capacity

The total antioxidant capacity was evaluated using the phosphomolybdenum method [40]. In this method, the formation of a green phosphate/Mo (V) complex for the reduction of Mo (VI) to Mo (V) by an antioxidant is measured at an acidic pH. To achieve this, 100 µL of extracts were combined with 1 mL of a solution (0.6 M sulfuric acid, 28 mM sodium phosphate, and 4 mM ammonium molybdate). Samples were incubated in a thermo-block at 95 °C for 90 min. The samples were subsequently cooled to room temperature, and the absorbance of each was measured at 695 nm using a PowerWave HT Microplate Spectrophotometer (BioTek Instruments, Inc., Winooski, VT, USA) against a blank formed by all components of the reaction mixture and adding absolute methanol instead of extract. An ascorbic acid standard curve (A_695_ = 0.5002 [ascorbic acid] − 0.0036, *R^2^* = 0.9845) was generated with seven concentrations of ascorbic acid (0.03–0.3 mg/mL). Total antioxidant capacity values were expressed as milligrams of ascorbic acid equivalents per gram of dry tissue (AAE/g DT).

### 3.6. Cell Culture, Cytotoxic Effect, and Reactive Oxygen Species (ROS) Assay

#### 3.6.1. Cell Lines 

K562 and HL60 hematologic neoplasia cells lines, purchased from American Type Culture Collection (ATCC), and normal mononuclear cells (NMC), obtained from a healthy individual were used. NMC were obtained according to the previously described methodology [41]. One hundred mL of peripheral blood was collected from a healthy donor (male and 36 years old). Blood (100 mL) was diluted 1:1 with phosphate-buffered saline (PBS) (Invitrogen, Grand Island, NY, USA) and overlaid onto an equivalent volume of Ficoll-paque Plus (1.077 ± 0.001 g/mL). Cells were centrifuged at 740 × *g* for 30 min at room temperature. NMC were isolated and washed twice with endothelial basal medium-2 (EBM-2) supplemented with 3% FBS, 10 ng/mL streptomycin/penicillin, and 0.25 μg/mL amphotericin B (complete EGM-2 medium). The number of viable nucleated cells was determined in a Neubauer chamber (Marienfeld, Germany) using Turk solution as a diluent and trypan blue, respectively. All cell lines were grown in RPMI-1640 culture medium added with 10% FBS and 1% antibiotic (streptomycin/penicillin) in 5% CO_2_ atmosphere at 37 °C [42].

#### 3.6.2. Cell Viability and Proliferation Assay

In order to determine the cytotoxic effect of the *A. linaria* extracts, the cell lines (2 × 10^5^) were plated in 24 well plates and exposed *in vitro* with different concentrations of dry extracts (25, 50, 75, 100, 150 µg/mL) diluted in 0.1% (*v*/*v*) DMSO for 48 h at 37 °C. Subsequently, the cells were washed using 1 mL of FACS Flow cytometry solution, and centrifuged at a speed of 423× *g* for 6 min at 19 °C. Then, the supernatant was decanted and 200 µL of FACS cytometry solution and 200 µL of DAPI fluorescent label (2 µL/mL) were added. After that, the cells were incubated for 15 min at the same conditions previously mentioned. Finally, the viability was evaluated in a BD FACSVerse flow cytometer (BD Biosciences, San Jose, CA, USA). In all cases, a negative control (without extract) and a positive control (with 2.5 µM Imatinib) were used. The value of the median inhibitory concentration (IC_50_) was calculated using the FlowJo software version 10 (Becton, Dickinson and Company, Franklin Lakes, NJ, USA).

#### 3.6.3. Selectivity Index

To measure the selective cytotoxicity of the extracts against cancerous cells and the safety of the extracts towards normal cells, the selectivity index (SI) was determined. Therefore, the extracts were tested for cytotoxicity in the NMC cell line. 

The SI was calculated using the following equation:

(3) $$\mathrm{SI}\;=\;{\mathrm{IC}}_{50}\;\mathrm{NMC}\;\mathrm{cells}\;/\;{\mathrm{IC}}_{50}\;\mathrm{cancer}\;\mathrm{cells}$$

#### 3.6.4. Reactive Oxygen Species (ROS) Assay

The reactive oxygen species (ROS) assay was performed exclusively on the HL60 cell line. A discrimination filter was applied to select the extracts that were analyzed by the ROS assay. The filter consisted of identifying those extracts that had a SI ≥ 5, a moderate to high concentration of phenolic compounds (7 to 10 mg GAE/g DT), moderate to high concentration of flavonoid compounds (2.5 to 4 mg CE/g DT), and those with an IC_50_ value ≤ 30 µg/mL of cytotoxic activity. Consequently, 9 extracts that showed the highest potential were analyzed.

The ROS assay consisted of applying the extract concentration (IC_50_ value) of each of the selected extracts after discrimination, obtained in the cell viability and proliferation test against the HL60 cell line. First, the cells were stained with 2 µL of 20 µM DCFDA reagent which oxidizes and fluoresces when it reacts with ROS and incubated for 30 min at 37 °C. Then, the cells were exposed in the absence (negative control) or presence (IC_50_ value) to the extracts at two different evaluation times: 1 and 3 h. Parthenolide at 7.5 µM (dissolved in DMSO) was used as a positive control. Subsequently, the production of ROS was evaluated with a BD FACSVerse flow cytometry (BD Biosciences, San Jose, CA, USA). Finally, the data obtained from the flow cytometry were analyzed with the FlowJo software version 10 (Becton, Dickinson and Company, Franklin Lakes, NJ, USA) and expressed as median fluorescence intensity (MFI).

### 3.7. Statistical Analysis

All determinations were performed in triplicate. Results are reported as the mean ± standard deviation of three independent replicates. Results were subjected to an analysis of variance (ANOVA). Differences between values with a *p* < 0.05 were considered significant for the phenolic contents and antioxidants determinations. Tukey’s test was performed for the comparison of means for the corresponding results. For the cell assays, the extracts concentrations were log-transformed (base 10) to normalize the data. Dunnett’s test was used to assess the statistical significance of differences between test and control data (*p* < 0.05 and *p* < 0.01). Relationships between all determinations were tested using Pearson’s correlation. These analyses were carried out using SPSS software version 25.0 (SPSS Inc, Chicago, IL, USA). 

## 4. Conclusions

The phenolic composition, antioxidant and cytotoxic activities of *A. linaria* leaves were determined. The phenolic composition of *A. linaria* leaves showed that the main compounds were flavonoids and phenolic acids. The recognized antioxidant flavonoids, rutin, and quercetin were identified in *A. linaria* leaves. In addition, the leaves extracts of *A. linaria* showed a high radical scavenging activity, high total antioxidant capacity values, and had cytotoxic effect against K562 and HL60 hematologic neoplasia cells lines, but no toxicity towards the normal mononuclear cell line was observed. It can be concluded that the *A. linaria* species has the potential to be a source of extraction of phenolic compounds with relevant antioxidant and cytotoxic activities, with applications in multiple research fields.

## Figures and Tables

**Table 1:** Total phenolic, flavonoids, and tannins contents, free radical scavenging activity, and total antioxidant capacity of methanolic extracts from leaves of wild populations of *Asclepias linaria*.

Extract	TPC (mg GAE/g DT)	TFC (mg CE/g DT)	CTC (mg CE/g DT)	ABTS^•+^	DPPH^•^	TAC (mg AAE/g DT)
				IC_50_ (mg/mL)		
P1-M100	7.17 ± 0.46 ^g^	3.19 ± 0.05 ^d,e,f^	1.67 ± 0.06 ^d^	0.82 ± 0.016 ^h^	0.16 ± 0.001 ^d^	63.78 ± 2.22 ^c^
P1-M50	9.32 ± 0.27 ^b,c,d,e^	2.68 ± 0.11 ^e,f^	0.65 ± 0.02 ^h^	0.53 ± 0.009 ^a^	0.12 ± 0.001 ^a^	17.96 ± 1.47 ^g^
P2-M100	8.77 ± 0.09 ^d,e^	4.02 ± 0.31 ^a,b^	2.30 ± 0.03 ^b^	0.74 ± 0.008 ^g^	0.16 ± 0.001 ^c,d^	74.47 ± 6.08 ^b^
P2-M50	9.76 ± 0.37 ^a,b,c^	3.21 ± 0.28 ^d,e,f^	0.85 ± 0.03 ^f,g,h^	0.67 ± 0.002 ^f^	0.15 ± 0.001 ^b,c^	60.56 ± 0.78 ^c,d^
P3-M100	8.68 ± 0.20 ^d,e^	3.92 ± 0.26 ^a,b,c^	1.63 ± 0.14 ^d^	0.85 ± 0.012 ^i^	0.16 ± 0.001 ^c,d^	75.89 ± 3.85 ^b^
P3-M50	10.49 ± 0.47 ^a^	2.62 ± 0.21 ^e,f^	0.81 ± 0.02 ^f,g,h^	0.51 ± 0.003 ^a^	0.18 ± 0.002 ^e,f^	49.29 ± 1.55 ^e^
P4-M100	8.38 ± 0.26 ^e,f^	2.87 ± 0.22 ^d,e,f^	3.16 ± 0.16 ^a^	0.68 ± 0.009 ^f^	0.19 ± 0.001 ^f^	78.74 ± 0.62 ^b^
P4-M50	10.50 ± 0.27 ^a^	2.56 ± 0.26 ^f^	0.68 ± 0.03 ^g,h^	0.59 ± 0.003 ^b,c^	0.15 ± 0.001 ^b,c^	60.82 ± 0.82 ^c,d^
P5-M100	9.07 ± 0.51 ^c,d,e^	4.17 ± 0.31 ^a^	2.05 ± 0.06 ^c^	0.60 ± 0.008 ^c,d^	0.15 ± 0.001 ^b,c^	81.24 ± 3.86 ^b^
P5-M50	10.10 ± 0.15 ^a,b^	3.28 ± 0.04 ^c,d,e^	1.00 ± 0.08 ^f^	0.58 ± 0.007 ^b,c^	0.16 ± 0.001 ^c,d^	36.08 ± 0.39 ^f^
P6-M100	7.48 ± 0.33 ^f,g^	3.38 ± 0.31 ^b,c,d^	1.22 ± 0.06 ^e^	0.75 ± 0.001 ^g^	0.18 ± 0.002 ^e^	99.77 ± 4.32 ^a^
P6-M50	9.58 ± 0.22 ^a,b,c,d^	2.94 ± 0.10 ^d,e,f^	0.88 ± 0.05 ^f,g^	0.64 ± 0.008 ^e^	0.14 ± 0.001 ^b^	29.61 ± 1.25 ^f^
P7-M100	8.66 ± 0.53 ^d,e^	3.24 ± 0.17 ^c,d,e^	1.46 ± 0.06 ^d^	0.62 ± 0.004 ^d,e^	0.15 ± 0.001 ^b,c^	95.49 ± 4.32 ^a^
P7-M50	10.44 ± 0.09 ^a^	3.09 ± 0.25 ^d,e,f^	0.43 ± 0.03 ^i^	0.57 ± 0.002 ^b^	0.15 ± 0.001 ^b,c^	54.99 ± 0.60 ^d,e^

Abbreviations: TPC, total phenolic content; TFC, Total flavonoid content; CTC, condensed tannin content; M100, extracted with 100% methanol; M50, extracted with 50% methanol; GAE, gallic acid equivalents; CE, catechin equivalents; AAE, ascorbic acid equivalents; DT, dry tissue; DPPH, 2,2-diphenyl-1-picrylhydrazyl; ABTS, 2,2-azino-bis[3-ethylbenzothiazoline-6-sulphonic acid]; IC_50_, median inhibitory concentration. Values are expressed as mean ± standard deviation of three repetitions. Values with different letters indicate significant differences (Tukey, *p* < 0.05).

**Table 2:** Phenolic composition of 50 and 100% methanol extracts of *Asclepias linaria* leaves identified by ultra-performance liquid chromatography coupled to mass spectrometry (UPLC-MS).

Extract		*p*-coumaric acid	Ferulic acid	Rutin	Quercetin	Total
P1-M100	*t*_R_ (min)	ND	6.29 ± 0.01	7.15 ± 0.01	8.90 ± 0.00	
	µg/mL	ND	0.49 ± 0.02 ^g^	37.53 ± 1.10 ^e^	11.33 ± 0.23 ^c,d^	49.35 ± 0.62 ^f,g^
P1-M50	*t*_R_ (min)	ND	6.27 ± 0.01	7.15 ± 0.00	8.87 ± 0.06	
	µg/mL	ND	0.40 ± 0.00 ^g,h^	30.00 ± 0.40 ^f^	13.47 ± 0.61 ^a,b,c^	43.87 ± 0.21 ^g^
P2-M100	*t*_R_ (min)	ND	6.29 ± 0.01	7.15 ± 0.01	8.91 ± 0.01	
	µg/mL	ND	0.82 ± 0.06 ^e^	49.33 ± 0.57 ^d^	6.00 ± 0.00 ^h^	56.15 ± 0.13 ^d,e^
P2-M50	*t*_R_ (min)	ND	6.29 ± 0.01	7.15 ± 0.00	8.91 ± 0.01	
	µg/mL	ND	0.72 ± 0.06 ^e,f^	41.33 ± 1.52 ^e^	10.00 ± 1.00 ^d,e,f^	52.05 ± 0.42 ^e,f^
P3-M100	*t*_R_ (min)	ND	6.28 ± 0.01	7.15 ± 0.00	8.90 ± 0.00	
	µg/mL	ND	0.96 ± 0.00 ^d^	28.00 ± 0.00 ^f^	5.17 ± 0.58 ^h^	34.13 ± 1.34 ^h^
P3-M50	*t*_R_ (min)	ND	6.28 ± 0.01	7.15 ± 0.01	8.91 ± 0.00	
	µg/mL	ND	0.34 ± 0.02 ^h^	25.67 ± 0.70 ^f^	8.40 ± 0.69 ^f,g^	34.41 ± 0.50 ^h^
P4-M100	*t*_R_ (min)	5.9 ± 0.01	6.27 ± 0.01	7.14 ± 0.00	8.90 ± 0.01	
	µg/mL	1.0 ± 0.00 ^a^	0.75 ± 0.00 ^e,f^	51.50 ± 0.00 ^d^	6.72 ± 0.96 ^g,h^	59.97 ± 2.83 ^d^
P4-M50	*t*_R_ (min)	ND	6.28 ± 0.02	7.15 ± 0.01	8.91 ± 0.01	
	µg/mL	ND	0.49 ± 0.00 ^g^	37.67 ± 0.76 ^e^	13.00 ± 0.50 ^b,c^	51.16 ± 0.32 ^e,f^
P5-M100	*t*_R_ (min)	5.89 ± 0.00	6.29 ± 0.03	7.15 ± 0.00	8.91 ± 0.01	
	µg/mL	0.25 ± 0.01 ^b^	0.68 ± 0.10 ^f^	61.33 ± 4.50 ^c^	9.00 ± 1.00 ^e,f^	71.26 ± 1.34 ^c^
P5-M50	*t*_R_ (min)	ND	ND	7.15 ± 0.00	8.90 ± 0.00	
	µg/mL	ND	ND	16.53 ± 1.00 ^g^	15.00 ± 0.60 ^a,b^	31.53 ± 0.76 ^h^
P6-M100	*t*_R_ (min)	ND	6.28 ± 0.01	7.15 ± 0.01	8.90 ± 0.01	
	µg/mL	ND	1.13 ± 0.01 ^c^	72.33 ± 1.52 ^b^	6.33 ± 0.58	79.79 ± 0.82 ^b^
P6-M50	*t*_R_ (min)	ND	ND	7.16 ± 0.01	ND	
	µg/mL	ND	ND	0.73 ± 0.05 ^h^	ND	0.73 ± 0.45 ^i^
P7-M100	*t*_R_ (min)	ND	6.31 ± 0.02	7.16 ± 0.01	8.99 ± 0.00	
	µg/mL	ND	1.90 ± 0.12 ^a^	79.67 ± 2.51 ^a^	10.67 ± 0.58 ^d,e^	92.24 ± 1.37 ^a^
P7-M50	*t*_R_ (min)	ND	6.28 ± 0.01	7.16 ± 0.01	8.91 ± 0.00	
	µg/mL	ND	1.58 ± 0.04 ^b^	76.67 ± 3.51 ^a,b^	15.67 ± 1.53 ^a^	93.92 ± 0.30 ^a^

Abbreviations: M100, extracted with 100% methanol; M50, extracted with 50% methanol; *t*_R_, retention time; ND, not determined. Values are expressed as the mean ± standard deviation of three repetitions. Values with different letters indicate significant differences (Tukey, *p* < 0.05)

**Table 3:** Anti-proliferative activity of 50 and 100% methanol extracts of *Asclepias linaria* leaves against hematologic neoplasia cells lines (K562 and HL60) and normal mononuclear cells (NMC).

Extract	K562 IC_50_ (µg/mL)	HL60 IC_50_ (µg/mL)	CMN IC_50_ (µg/mL)	SI K562	SI HL60
P1-M100	72.13 ± 7.59 ^b^	17.33 ± 0.43 ^d^	116.7 ± 13.57 ^c,d,e,f^	1.61	6.73
P1-M50	28.22 ± 4.09 ^a^	16.79 ± 0.86 ^c,d^	18.7 ± 0.48 ^a,b^	0.66	1.11
P2-M100	79.57 ± 10.98 ^b^	14.25 ± 0.85 ^b,c^	155 ± 1.94 ^d,e,f^	1.94	10.88
P2-M50	12.05 ± 1.60 ^a^	11.31 ± 0.15 ^a^	33.6 ± 5.07 ^a,b,c^	2.77	2.97
P3-M100	29.40 ± 2.03 ^a^	14.28 ± 0.94 ^b,c^	66.8 ± 7.14 ^a,b,c,d^	2.27	4.68
P3-M50	153.47 ± 28.77 ^c^	17.53 ± 1.41^d^	187.3 ± 43.56 ^f^	1.22	10.68
P4-M100	18.78 ± 2.95 ^a^	15.54 ± 0.42 ^b,c,d^	100.4 ± 8.95 ^b,c,d,e,f^	5.34	6.46
P4-M50	167.42 ± 24.03 ^c^	16.00 ± 0.89 ^b,c,d^	130.3 ± 10.74 ^d,e,f^	0.77	8.14
P5-M100	>400 ^d^	15.50 ± 0.83 ^b,c,d^	ND	ND	ND
P5-M50	15.45 ± 0.56 ^a^	20.89 ± 1.59 ^e^	108.3 ± 15.49 ^c,d,e,f^	7.0	5.18
P6-M100	10.07 ± 0.08 ^a^	17.45 ± 1.34 ^d^	96.8 ± 4.88 ^b,c,d,e^	9.58	5.55
P6-M50	8.96 ± 0.29 ^a^	13.52 ± 0.87 ^a,b^	184.2 ± 13.19 ^e,f^	20.46	13.62
P7-M100	9.52 ± 1.28 ^a^	11.27 ± 0.27 ^a^	155 ± 13.91 ^d,e,f^	16.31	13.75
P7-M50	133.55 ± 14.36 ^c^	11.10 ± 0.48 ^a^	244.9 ± 96.16 ^f^	1.80	22.06

Abbreviations: M100, extracted with 100% methanol; M50, extracted with 50% methanol; SI, selectivity index; IC_50_, median inhibitory concentration; > 400, IC_50_ value greater than 400 µg/mL. ND, not determined. Values are expressed as mean ± standard deviation of three repetitions. Values with different letters indicate significant differences (Tukey, *p* < 0.05). Two independent trials were performed in duplicate.

**Table 4:** Mass spectrometric conditions for each compound.

Compound	*t*_R_ (min)	Ion Mode	MRM (*m*/*z*)	Cone (V)	Collision (eV)
Vanillin	5.46	+	153.01 ˃ 125.03	40	0
(+)-Catechin	3.97	+	291.15 ˃ 123.10 291.15 ˃ 165.10	13 13	13 10
*p*-coumaric acid	5.84	-	163.10 ˃ 119.00	20	10
Gallic acid	1.45	-	169.10 ˃ 125.00	15	12
Caffeic acid	4.61	-	179.10 ˃ 135.00	20	10
Ferulic acid	6.53	-	193.07 ˃ 133.90 193.07 ˃ 178.04	10 10	13 17
Quercetin	8.86	-	301.02 ˃ 150.99 301.02 ˃ 178.98	20 20	10 10
Naringin	9.11	+	273.20 ˃ 147.10 273.20 ˃ 153.05	35 35	20 20
Apigenin	9.97	-	269.15 ˃ 117.00 269.15 ˃ 151.00	30 30	37 23
Rutin	7.15	-	609.10 > 300.0	80	28
Chlorogenic acid	4.26	-	353.05 > 191.03	20	15
Cinnamic acid	9.01	-	146.80 > 103.00	10	10
